# Clinically Unusual Pigmented Lesion of the Buccal Mucosa: A Case Report

**DOI:** 10.7759/cureus.45050

**Published:** 2023-09-11

**Authors:** Yanko G Yankov, Anna K Nenova-Nogalcheva, Simeon N Dimanov, Lyuben L Stoev, Desislava A Konstantinova

**Affiliations:** 1 Department of General and Operative Surgery, Medical University "Prof. Dr. Paraskev Stoyanov", Varna, BGR; 2 Department of Oral Surgery, Medical University of Varna, Varna, BGR; 3 Department of General and Clinical Pathology, Forensic Medicine and Deontology, Medical University of Varna, Varna, BGR; 4 Department of Dental Material Science and Prosthetic Dental Medicine, Medical University of Varna, Varna, BGR

**Keywords:** pigment induced disease, tattoo pigment, mucosal disease, oral mucosal lesions, oral and maxillofacial pathology, tattoo, pigmentation, oral and maxillofacial surgery, mouth mucosa, dental amalgam

## Abstract

We present a rare clinical case of a 64-year-old patient with a pigmented lesion localized in the left buccal mucosa. Subjective complaints of slight pain and discomfort in the process of eating and increased sensitivity when consuming hot food were reported. According to the information provided by the patient, the lesion had progressively increased in size. A history of previous dental manipulations was reported, namely, the extraction of teeth with amalgam obturations in the left half of the maxilla as per relevant indications. During the intraoral examination, a flat, black-colored lesion, 0.4 cm in diameter, with well-defined borders was observed in the buccal mucosa. Teeth 25, 26, and 27 were previously extracted five to seven years ago. An orthopantomography was performed as a routine procedure. It did not show any presence of X-ray contrast areas that could explain the symptoms of the patient. The symptomatic nature of the lesion as well as the negative radiological findings prompted surgical treatment and excisional biopsy with subsequent histological evaluation to rule out oral malignancy. An excision was performed. During the follow-up examination in the next eight days, all the symptoms of the patient were gone. The conclusion of the pathology report was "histological findings and clinical data consistent with amalgam tattoo". The amalgam tattoo is the most frequent iatrogenic pigmented lesion of the oral mucosa, which results from the implantation of amalgam particles in the soft tissues and it is usually asymptomatic. In this case, no surgical treatment is needed. However, in some rare cases, like the one we are presenting, some symptoms can occur and complicate the diagnostic process. In these cases, the complete excision of the lesion is to be performed with subsequent histological evaluation. The atraumatic intervention of teeth, obturated with definitive amalgam fillings, is a main factor for preventing this kind of pigmentation of the oral mucosa.

## Introduction

The term “pigmentation of the oral mucosa” covers a wide range of conditions or lesions related to the change of the color of the oral tissues. In their research from 2008, Meleti et al. classified the pigmentations into endogenous and exogenous according to their etiology. Endogenous pigmentations include melanin, hemoglobin, hemosiderin, and carotene, which are deposited in the soft tissues in various ways. Exogenous pigmentations are most often related to the implantation of foreign bodies in the oral mucosa [1].

The amalgam tattoo is one of the most frequent reasons for intraoral exogenous pigmentations. According to different research, the incidence of amalgam tattoos in pigmented oral lesions can vary greatly, between 0.4% and 0.9% for the adult population in the USA and 8% in Sweden [2]. This type of pigmentation is caused by the penetration of the amalgam in the soft tissues in case of inadvertent application of the material in the prepared cavity or during its removal after trauma or compromised integrity of the oral epithelium of the neighboring tissue caused by the high-speed nozzle. It is also possible that amalgam particles enter the surgical wound during tooth extraction; or in the case of endodontic treatment with retrograde filling, amalgam particles may be introduced in the soft tissues due to the lack of caution [3,4].

The amalgam pigmentation is most frequently localized on the gingiva and alveolar mucosa, but the lesions could also be found on the buccal mucosa, on the floor of the oral cavity, on the palate, and/or on the tongue. The usual clinical finding is of a localized, flat, gray and blue lesion with greatly variable size (2-19 mm). Usually, there are no signs of inflammation on the lesions’ periphery. In some cases, if the amalgam particles are sufficiently big, they can be found with the use of an X-ray. In this case, the diagnosis of amalgam tattoo will be based on the clinical and X-ray findings. In case of doubt, in order to confirm the diagnosis, an excisional biopsy is done [1,5-7].

## Case presentation

**Figure 1:**
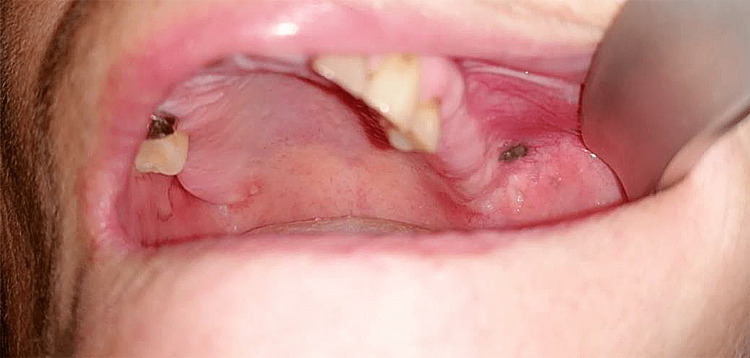
A 64-year-old female patient; intraoral view of the pigmented lesion

A 64-year-old female patient without concomitant diseases visited the Department of Oral Surgery, DDM - Varna. She had noticed a discoloration on the buccal mucosa about a month ago - a pigmented lesion in the area of the left buccal mucosa, and had subjective complaints of slight pain and discomfort while eating and increased sensitivity when consuming hot food. According to the information provided by the patient, the formation has progressively changed in size. A history of previous dental manipulations was reported, namely, the extraction of teeth with amalgam obturations in the left half of the maxilla as per relevant indications. During the intraoral examination, a flat, black-colored lesion, 0.4 cm in diameter, with well-defined borders was observed on the buccal mucosa. Teeth 25, 26, and 27 were previously extracted five to seven years ago (Figure 1).

**Figure 2:**
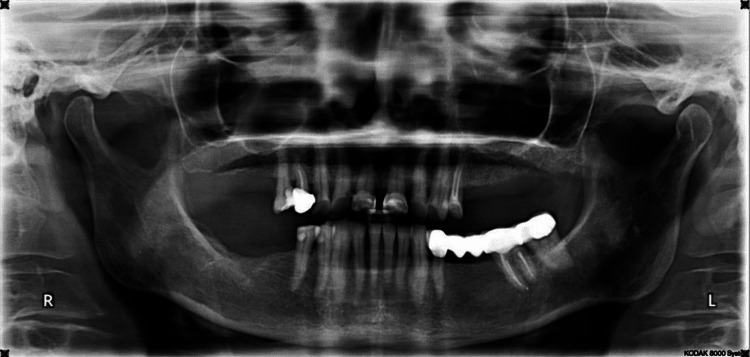
A 64-year-old female patient; orthopantomography

As a routine procedure, an orthopantomography was made. It did not show the presence of X-ray contrast areas that could explain the symptoms of the patient (Figure 2).

A consultation with a maxillofacial surgeon was conducted. The symptomatic nature of the lesion as well as the negative radiological findings for contrast areas prompted a surgical treatment and excisional biopsy with subsequent histological evaluation to rule out oral malignancy.

The surgical treatment was undertaken after a thorough antiseptic of the operative field using a Braunol solution. Local infiltrating anesthesia was applied with 0.5 ml Ubistesin forte. Using a leaf incision around the lesion of the mucosa in the left buccal area, the latter was excised within clear gross surgical margins. It was fixed in a 10% solution of formalin and sent for histological examination. After a thorough hemostasis, the wound was sutured with 4/0 non-resorbable monofilament.

**Figure 3:**
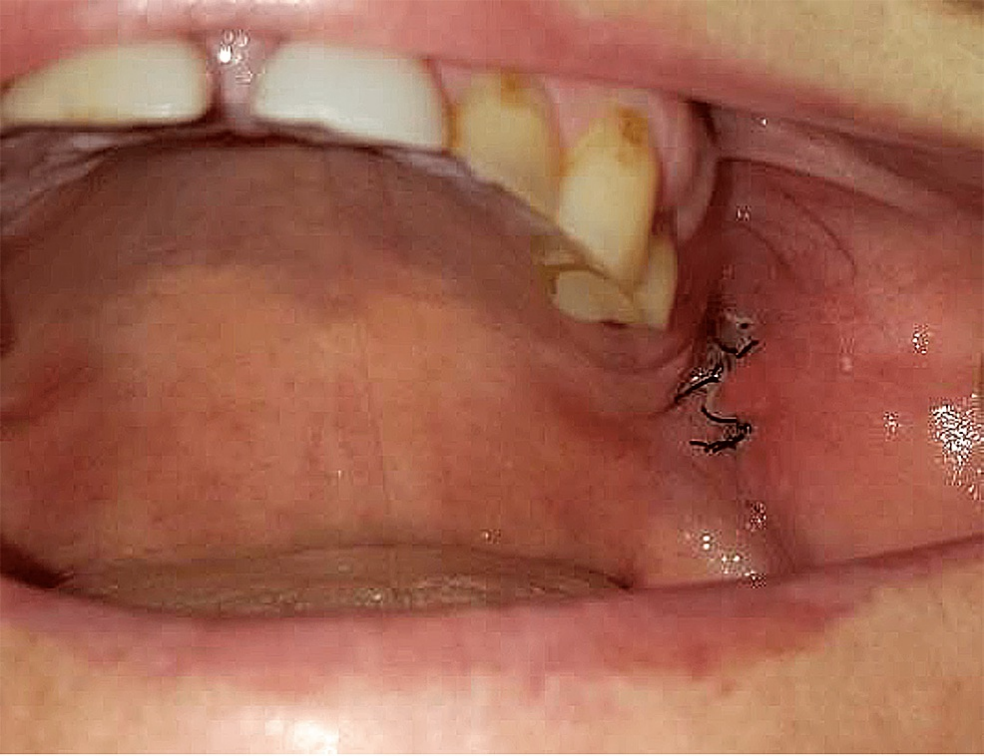
Intraoral view eight days after the operative treatment

Postoperatively, at home, the wound was treated by the patient with Listerine locally, three to four times a day. The postoperative period was smooth, without swelling and complaints (Figure 3). During the follow-up examination, the wound was stable and without bleeding. All the symptoms of the patient were gone. The sutures were removed on the eighth postoperative day.

**Figure 4:**
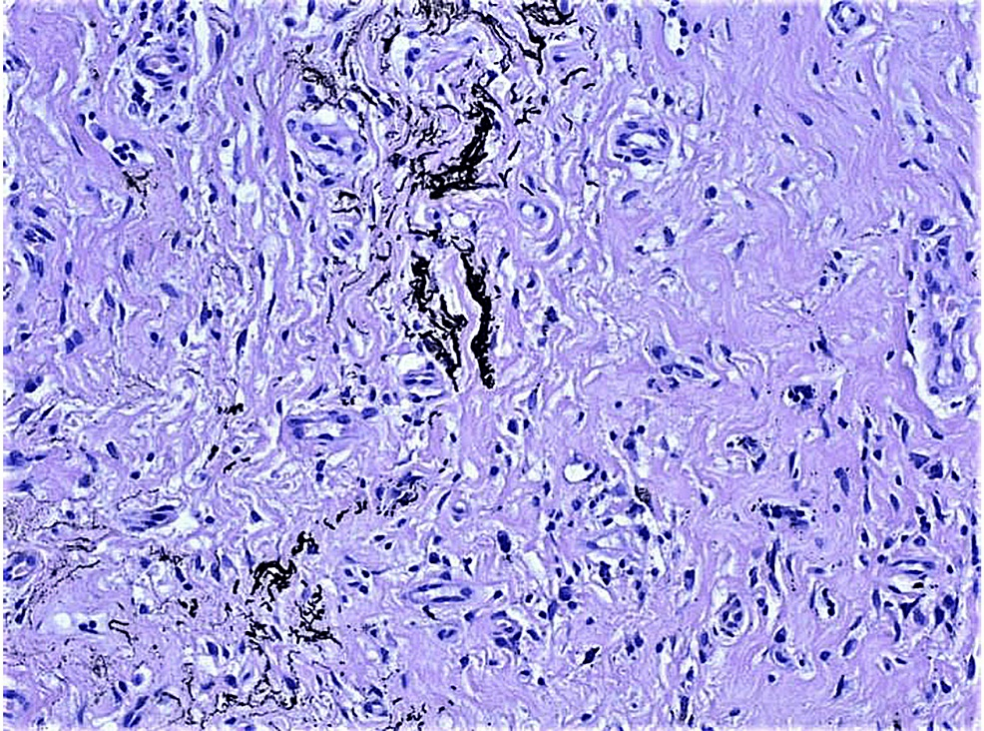
Black pigment in the interstitial matrix of the oral mucosa; scattered, single perivascular lymphocytes (Hematoxylin and Eosin, х 40)

The histological examination revealed the presence of single, scattered perivascular lymphocytes and scarce black extracellular pigment in the lamina propria. The pigment had no association with cellular elements like nevus cells and was absent in the overlying squamous epithelium. The conclusion of the pathology report was "histological findings and clinical data consistent with amalgam tattoo" (Figure 4).

During the follow-up period of six months, which the patient describes as uneventful, none of the symptoms occurred again.

## Discussion

Pigment lesions in the oral cavity can be divided into physiological and pathological. Physiological ones include only ethnic pigmentation. All other pigment colors are pathological and are divided into exogenous and endogenous. Exogenous pigments are drug-induced lesions, tobacco-chewed/smoking pigmentations, heavy metals induced, and amalgam tattoos. Endogenous are lesions caused by endocrine disorders (Addison's disease, hyperthyroidism, diabetes, and pregnancy), the syndrome associated (like neurofibromatosis and hemochromatosis), infectious agents (HIV, candidosis, and tuberculosis), posttraumatic and postinflammatory chronic irritation, reactive lesions (oral melanocytic macule and oral melanoacanthoma), and neoplastic lesions (benign nevus and malignant melanoma) [8].

According to another classification, there are two groups of pigmented lesions of the oral cavity depending on the type of the pigment that causes them - melanin-associated lesions, which refer to the melanin pigment (racial pigmentation, melanocytic macules, melanocytic nevus, and malignant melanoma) and lesions caused by other pigments (metal, amalgam, and blood pigmentations) [9].

Physiological (ethnic) pigmentation is related to greater melanocyte activity. Development is usually in the first two decades of life and is common for the African, Asian, and Mediterranean populations. Intraorally, the clinician can notice pigmentations that vary from light to dark brown and are located on the attached gingiva, buccal mucosa, hard palate, lips, and tongue. These lesions are asymptomatic and appear as a well-demarcated, ribbon-like band that can spread to the marginal gingiva. No treatment is required [1,4].

Melanocytic macules are benign lesions, most commonly located on the lower lip. They are caused by increased melanin production without an increase in the number of melanocytes. Melanotic macules are present with smooth, well-demarcated borders and are usually small in diameter, below 1 cm. The color varies from light to dark brown and is homogeneous within each lesion. As they are of benign nature, the treatment of these lesions includes excisional biopsy and histological examination. Once the diagnosis is confirmed, no further treatment is required. These lesions are not known to transform into melanoma [1,8].

Melanocytic nevi are lesions most commonly present on the buccal mucosa. Their color is brown or blue. They can be classified histologically as junctional, intramucosal, or compound nevi. The intramucosal is the most common lesion and is typically light brown in color and dome-shaped. The clinical differentiation between a nevus and an early form of mucosal melanoma may be difficult if the location is the palate. Transformation of oral pigmented nevi to melanoma has not been well-documented. It is believed that melanocytic naevus may be a precursor lesion to oral melanoma. The recommended treatment plan is again an excisional biopsy and histological examination [1,3,6].

Malignant melanoma is a rare lesion on the oral mucosa and constitutes less than 1% of all oral malignancies. Usually, malignant melanoma lesions are found on the palate but can be present on other oral mucosal sites. Clinically, these lesions are most commonly asymptomatic and slow-growing, their color is brown or black, and their borders are asymmetric and irregular. Oral melanomas tend to grow rapidly, and ulceration, bleeding, pain, and bone destruction may be present. Some of these lesions may be amelanotic. The guideline for treatment is radical surgical excision with histologically verified clear margins. Chemotherapy and radiation therapy are ineffective, which means that the early detection of oral melanoma is key for a better prognosis [1,4,5].

Blood pigmentations in the oral mucosa can be induced by the accumulation and degradation of hemoglobin to biliverdin and bilirubin. Extravasation of blood is the cause and is commonly associated with trauma. Clinically, the lesions are red to black in color and not painful. Treatment is not required if the lesions are not infected [6,8].

The increased levels of heavy metals (lead, bismuth, silver, mercury, arsenic, and gold) in the blood are known as causes for changes in the color of the oral mucosa. In adult patients, the most frequent cause for such increased levels is exposure to heavy metal vapors. The pigmentations are clinically observed as a black and blue line around the border of the gingiva. Depending on the type of heavy metal, many systematic symptoms and conditions can be related to chronic exposure. The most important aspect in order to prevent systematic toxic complications in the case of oral mucosal pigmentation is the identification and treatment of the main cause [1,7].

Dental amalgams are the oldest obturation material, with over 150 years of history. Depending on the sputtering technology that determines the form of the particles in the powder, there are two types of amalgam alloys - lathe-cut and spherical. Depending on the components, they are classic and have high copper content (without ℽ 2-phase). Due to their good biological tolerance and mechanical qualities, the amalgam obturations of age over 25-30 years are frequently found during intraoral examination and registration of the tooth status for patients in Bulgaria. The increased esthetic requirements of the patients in the last years led to their replacement with composite obturation materials in some cases. In other cases, the occurrence of secondary carious foci as a result of the volume increase and deformation of the amalgam during chewing leads to complications such as fracturing of the enamel edges or whole walls of the clinical tooth crown, deformation of the amalgam at approximal contacts of the obturation with the filling of the interdental spaces, and other complications, which may show indications for tooth extraction. All described conditions are potential etiological factors for the occurrence of a pigmented lesion. With the establishment of the diagnosis amalgam tattoo, additional treatment is not required in general cases [10]. Follow-up examinations of the patient in six-month periods are enough, excluding cases where there are clinical symptoms or cosmetic indications when the lesion is localized in the esthetic area of the face [11].

In their research, Batra et al. present a clinical case of pigmentation of the gingiva in the area of the lower front teeth of a 45-year-old patient. The pigmented lesion is a result of retrograde filling with amalgam during apical osteotomy in the same area, performed four years earlier. The lesion localization in the aesthetical area requires excising and subsequent histological verification [12].

This case described by us is unusual because the leading factor for directing the operative treatment was the subjective complaint of pain, the progressive increase of the lesion size, as well as the negative radiological findings for contrast areas in orthopantomography. Pain as a clinical symptom is not typical for pigmented intraoral lesions [7,10,12]. In 2010, Parizi et al. described a case similar to ours, with symptoms of pain from the patient. The authors report chronic sinusitis associated with an amalgam tattoo in a 46-year-old woman. The presence of a contrasting X-ray lesion with a 2 mm diameter in the right oral mucosa was found on the panoramic X-rays and clinically observed as a black macule. After the histologic examination, it was verified as an amalgam-pigmented lesion. One year after the lesion removal, the patient informed that the complaints had ceased after the metal removal [13]. The increase in the size of the lesion can be due to either mechanical spread in the soft tissues or lymphatic transport. The latter is also typical for malignant melanoma. Each pigmented lesion in the oral cavity should be treated with the utmost caution, as there are no reliable definitive clinical criteria to tell apart malignant melanoma from benign melanocytic lesions or exogenous pigment deposition [14]. Furthermore, due to the lymphatic transport of metallic material, the discoloration can increase in size giving the impression of a growing malignant neoplasm [14]. Therefore these lesions should be excised within histologically verified clear margins, with the required for the purpose resection margin. According to our practice for forming, polishing, and removing amalgam obturation, an insulation sheeting, water-air spray, and aspiration system are to be used. For surgical interventions of teeth with amalgam obturations, atraumatic work, and good aspiration prevent undesired pigmented complications.

## Conclusions

Most frequently, the amalgam tattoos in the mucosa of the oral cavity are not a pathological finding. However, there are very rare symptomatic cases, like the one we are presenting in this case report, and their prognosis after surgical treatment is good. Each case of a pigmented lesion should be approached individually with great care due to the difficulties in the differential diagnosis of malignant lesions, even when the assessment is performed by an experienced clinician.
